# Supplementary material for: Plasmepsin X activates the PCRCR complex of Plasmodium falciparum by processing PfRh5 for erythrocyte invasion
Source: Nat Commun. 2023 Apr 19;14:2219. doi: 10.1038/s41467-023-37890-2 (PMC10113190; doi:10.1038/s41467-023-37890-2)
Supplement: Supplementary file 1 — Supplementary Information [file 41467_2023_37890_MOESM1_ESM.pdf]

Supplementary Information

Supplementary Figures

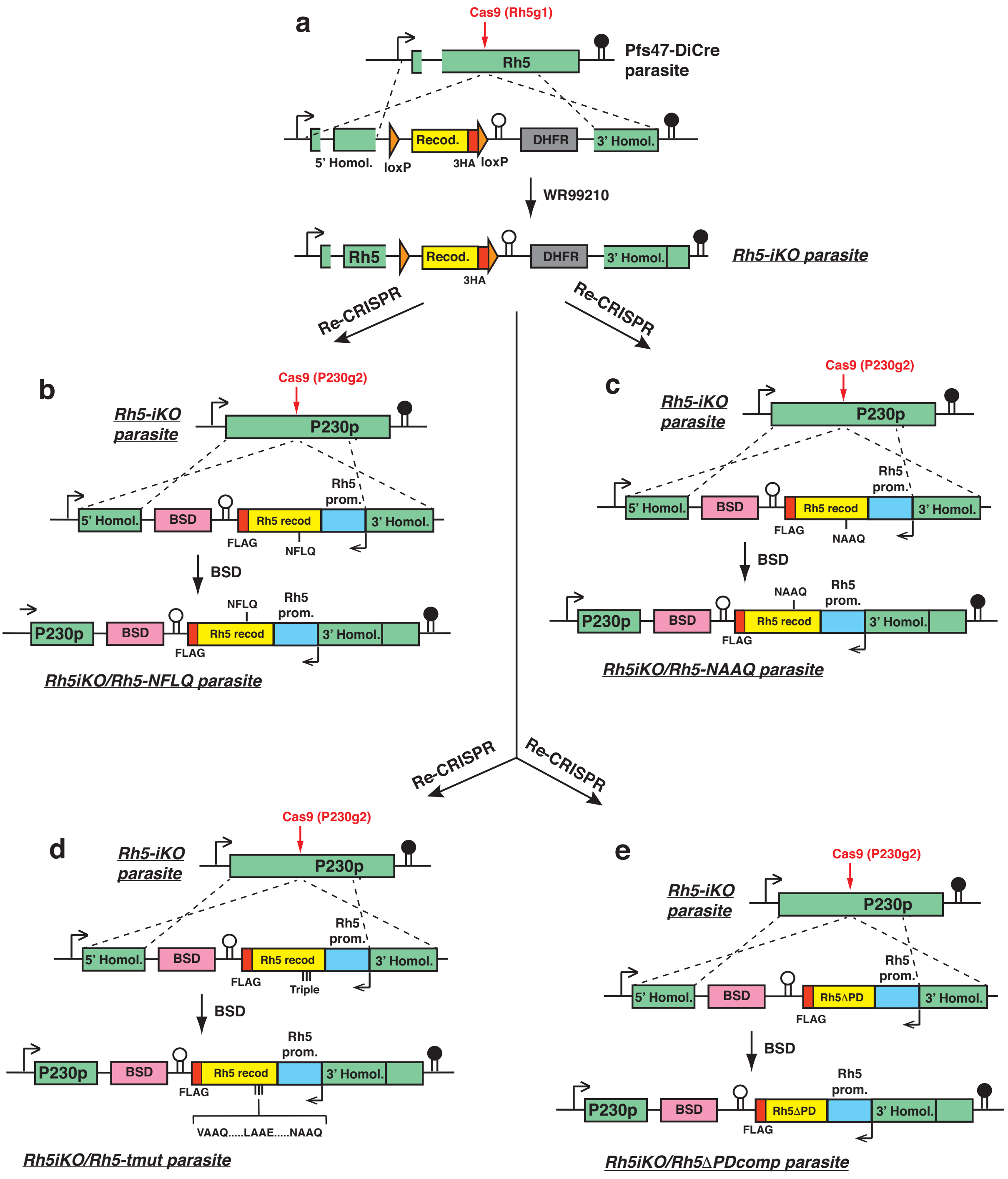

Figure S1. Constructs for complementation of the Rh5iKO parasite. a. The Rh5-iKO parasite was made in the Pfs47DiCre line that inducibly expresses the DiCre components, under the action of RAPA [1]. The 5' *loxP* site was introduced in a *sera5* intron, while the 3' *loxP* site was part of the plasmid. The right-pointing arrow is the endogenous *PfRh5* promoter,

the black filled-in stalk depicts the endogenous *PfRh5* terminator and the unfilled stalk depicts the *P.berghei dhfr-ts* terminator. **b.** A double-CRISPR strategy was used to generate a control parasite that can be PMX-cleaved at both the NFLQ site and at the alternative cleavage sites in the *P230p* locus, in the Rh5-iKO parasite (Rh5iKO/Rh5-NFLQ) that would enable complementation. In the *P230p* locus, the *PfRh5* gene was driven by a ~1 kb PCR-amplified *PfRh5* promoter sequence. The PfRh5 protein was FLAG-tagged and the selectable marker for the *P230p* locus was the blasticidin deaminase (*bsd*) gene. The *P230p* gene is dispensable in asexual stage parasites. **c.** A double-CRISPR strategy was used to generate a parasite expressing a PfRh5 protein that can only be PMX-cleaved at the alternative cleavage sites (Rh5iKO/Rh5-NAAQ), as the preferred cleavage site was mutated to NAAQ from NFLQ. **d.** A double-CRISPR strategy was used to generate a parasite expressing a PfRh5 protein containing three mutations at both the preferred site (NAAQ) and at both alternative sites (VAAQ and LAAE) to give the Rh5iKO/Rh5-tmut parasite. **e.** A double-CRISPR strategy was used to generate a parasite expressing a PfRh5 protein lacking its prodomain (Rh5ΔPDcomp).

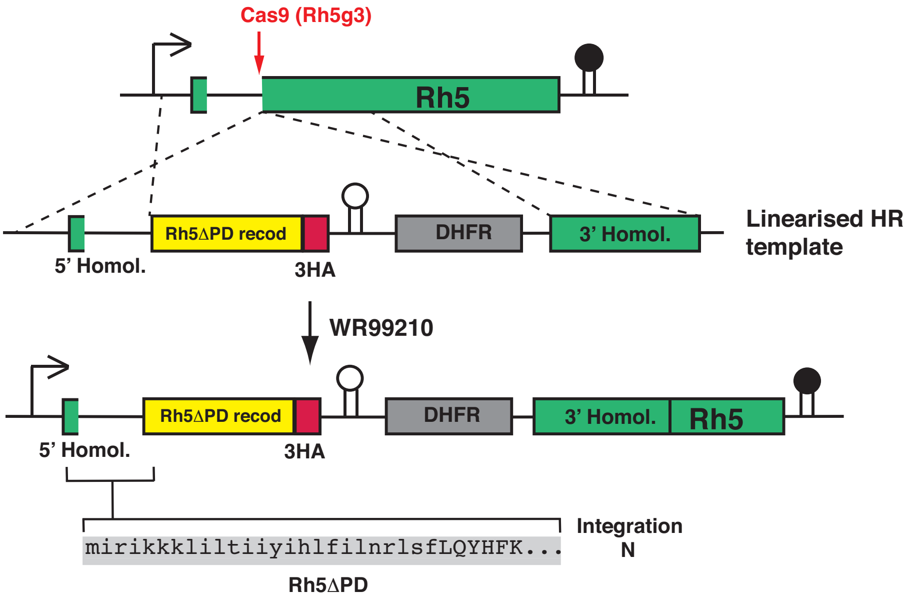

Figure S2. The PfRh5 prodomain (PD) is essential for parasite viability.
3D7 parasites were transfected with both a *cas9-Rh5g3* guide plasmid and a plasmid containing a recodoned sequence in a Homology Repair plasmid (HR template) that would produce a parasite lacking the PfRh5 PD. The encoded protein would contain the PfRh5 signal sequence (lower case) fused to the mature PMX-cleaved PfRh5 sequence (upper case), as shown in the lower panel. No viable parasites were obtained despite multiple attempts. The right-pointing arrow depicts the endogenous *pfrh5* promoter, and the black filled-in stalk depicts the endogenous *pfrh5* terminator. The unfilled stalk depicts the *P.berghei dhfr-ts* terminator.

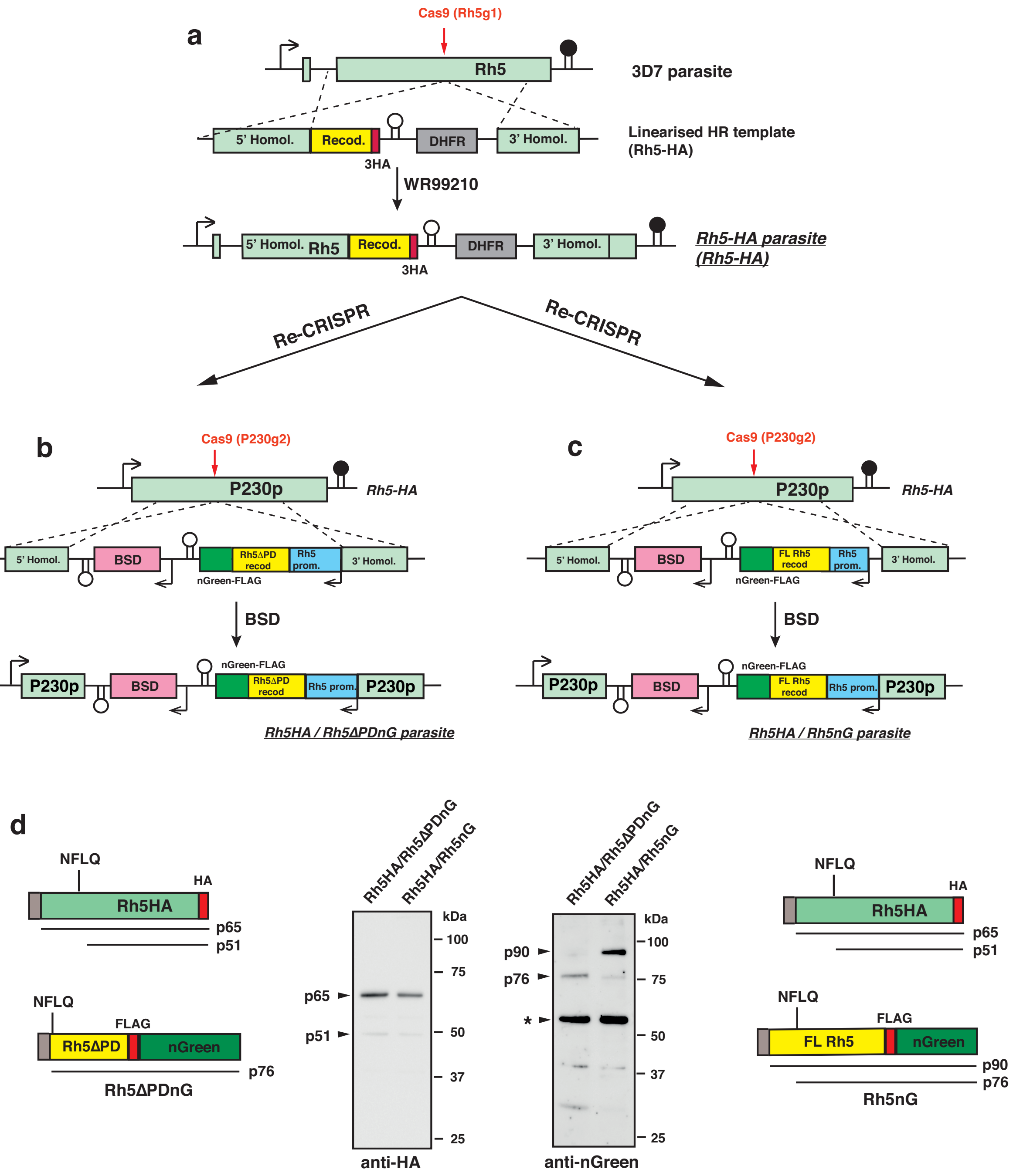

Figure S3. Transfectants expressing Rh5DPDnG and Rh5nG in addition to Rh5HA. a. The Rh5HA parasite has been previously described in Supplementary Fig.2. **b, c.** The Rh5HA parasite was used as the basis to express either the Rh5ΔPD or full-length (FL) Rh5 proteins tagged with nGreen-FLAG, from the *P230p* locus. The Rh5ΔPD or full-length (FL) Rh5 proteins were driven by a ~1 kb PCR-amplified *PfRh5* promoter sequence and BSD was used as selectable marker in the *P230p* locus. **d.** Proteins from synchronised schizonts of the Rh5HA/Rh5ΔPDnG and Rh5HA/Rh5nG parasites were blotted, then probed with anti-HA and anti-nGreen mAbs. Schematics for the expected proteins to be produced in each transfectantare shown to the sides of the immunoblots. Both transfectants show unprocessed (p65) and PMX-processed (p51) proteins when probed with anti-HA mAbs. When probed with anti-nGreen mAbs however, the Rh5HA/Rh5ΔPDnG parasite shows a p76 band, while the Rh5HA/Rh5nG

parasite shows both the unprocessed (p90) and PMX-processed (p76) bands. The cross-reacting protein is marked with an asterisk.

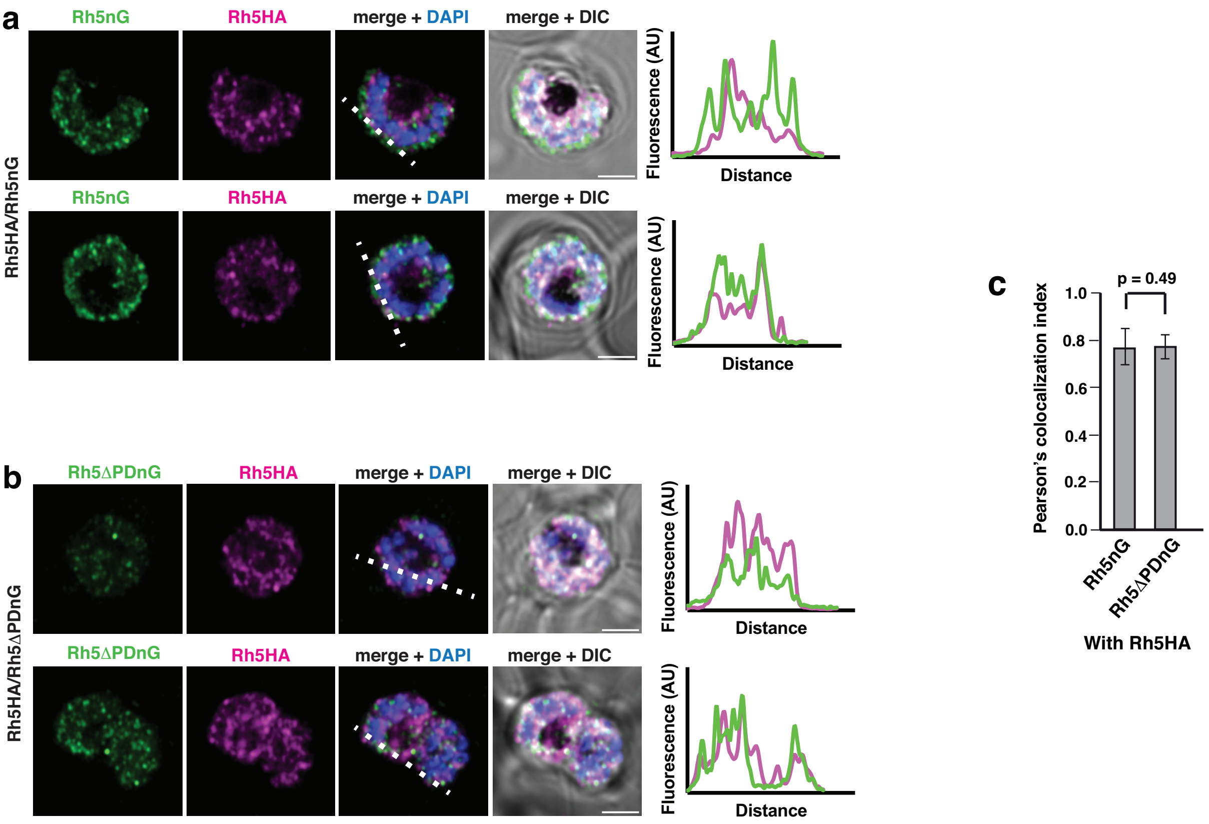

Figure S4. The Rh5PD has no role in PfRh5 trafficking. a. The Rh5PD is not required for post-Golgi trafficking to an apical organelle. Super-resolution images showing colocalization of Rh5HA and Rh5nGreen in late schizonts. Intensity plots along the white broken line show colocalization in the apical organelles. Scale bar 2 µm. **b.** Super-resolution images showing colocalization of Rh5HA and Rh5DPDnGreen in late schizonts. Intensity plots along the white broken line show colocalization in the apical organelles. Scale bar 2 µm. **c.** Pearson's colocalization index determined for 10 schizonts of each transfectant. Mean values are shown together with error bars representing standard deviation. Students t-test was used to calculate the p-value.

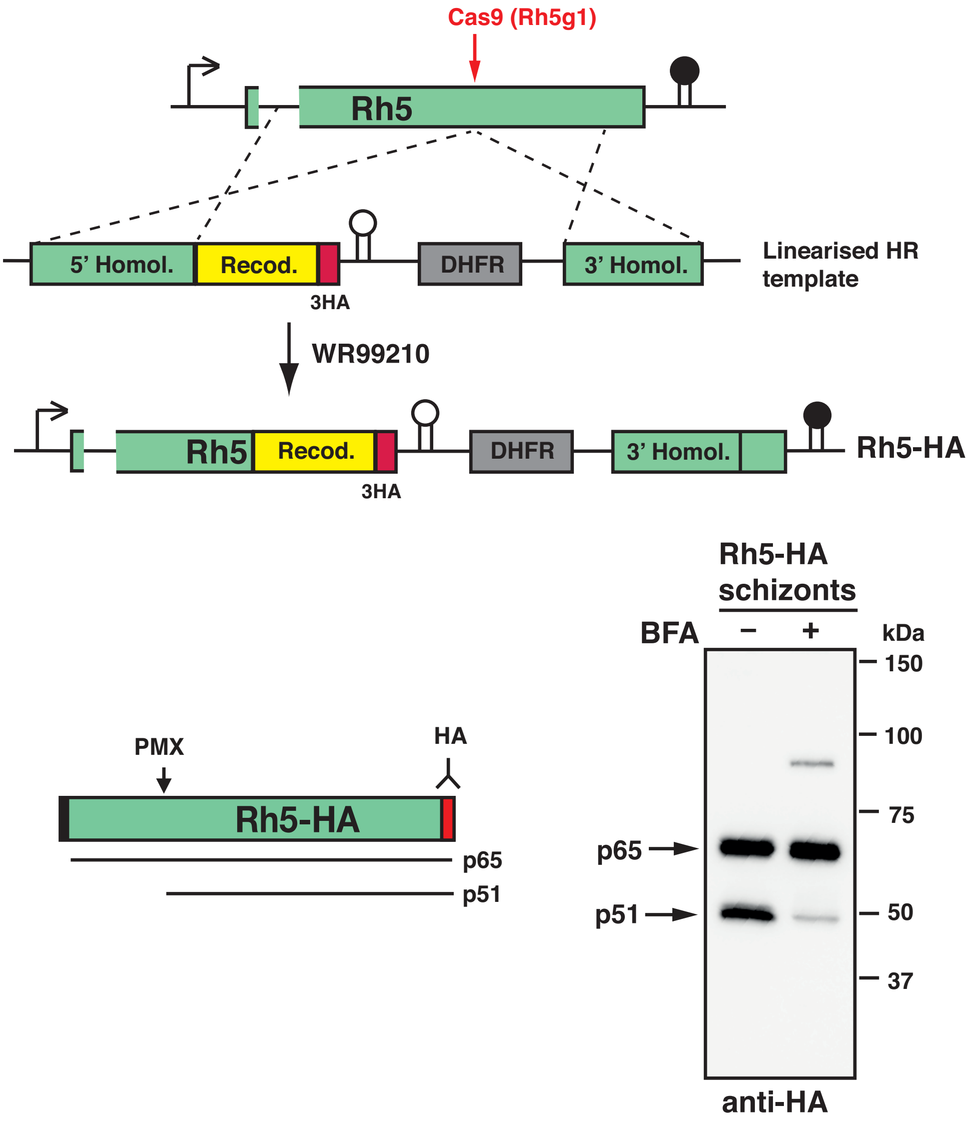

Figure S5. PfRh5 is processed by PMX in a post-Golgi compartment. A parasite with a C-terminally HA-tagged PfRh5 (Rh5-HA) was obtained using CRISPR by a similar strategy to the one described in Supplementary Fig. 1 and Materials and Methods. The schematic for the Rh5-HA protein is shown together with the PMX cleavage site and the expected protein products: the unprocessed protein with a predicted MW of 65 kDa (p65) and the PMX-processed product (p51), when probed with anti-HA Abs. Rh5-HA parasites were synchronised, grown to the schizont stage, then magnet-purified. Cultures were split into untreated and brefeldin A (BFA) cultures and treated for 4 hr with 0.5 µg/ml BFA. Schizont proteins were extracted in Reducing Sample Buffer, separated by SDS-PAGE and probed with anti-HA Abs.

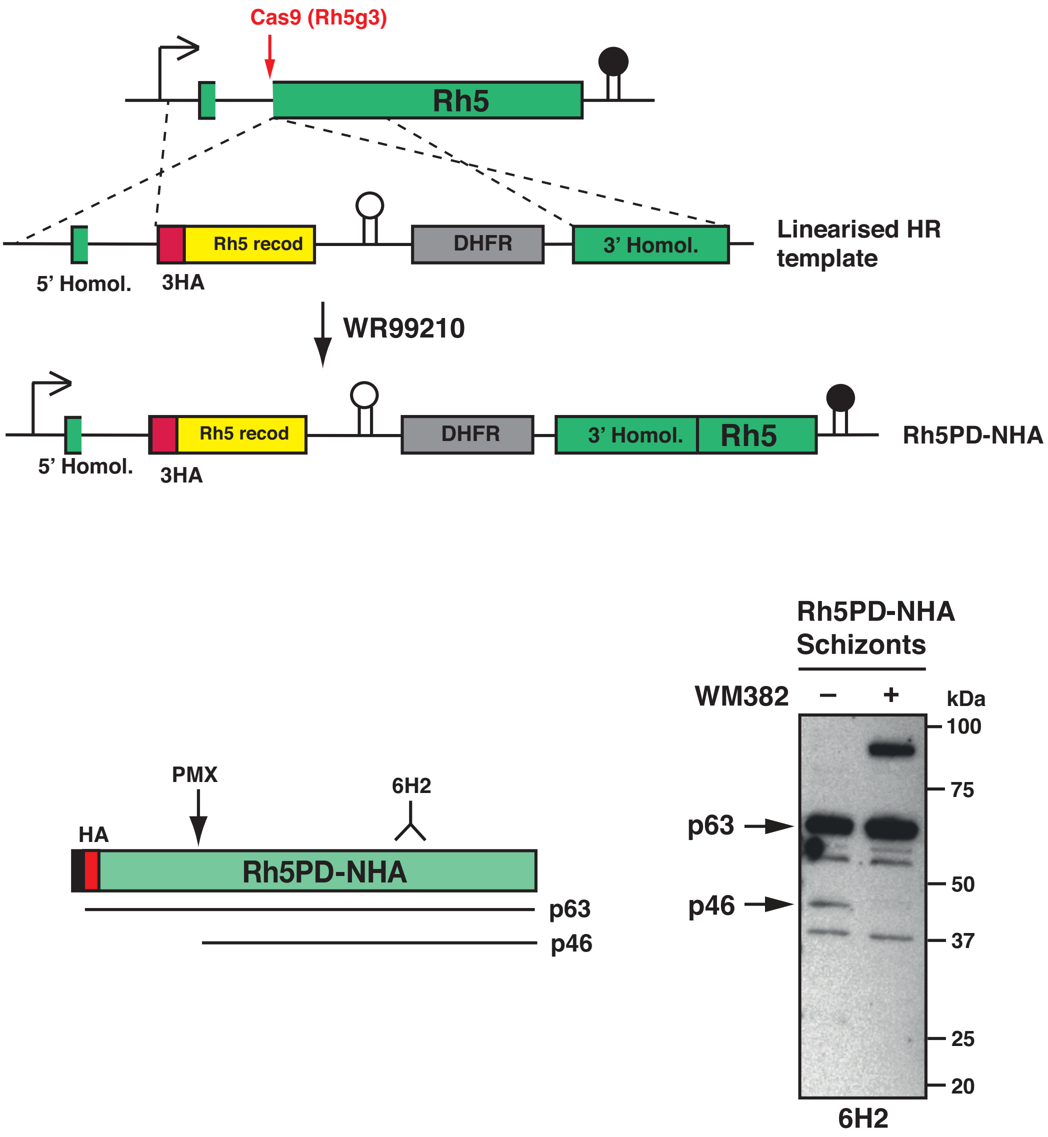

Figure S6. The Rh5 prodomain can be N-terminally tagged with HA. A parasite with a N-terminally HA-tagged prodomain (Rh5PD-NHA) was obtained using CRISPR by a strategy as described in Extended Data Fig. 1. The HA tag was fused to the PfRh5 signal sequence, and the resulting protein is shown in the schematic below. The unprocessed protein is expected to be 63 kD (p63) and the PMX-processed product is expected to be 46 kD, if detected with a PfRh5-specific mAb, 6H2. In the presence of the dual PMIX/PMX inhibitor WM382, the PMX processing will not occur, thus leaving the prodomain still attached to the mature PfRh5 protein. The western blot at right shows schizont proteins with and without WM382 probed with the 6H2 mAb.

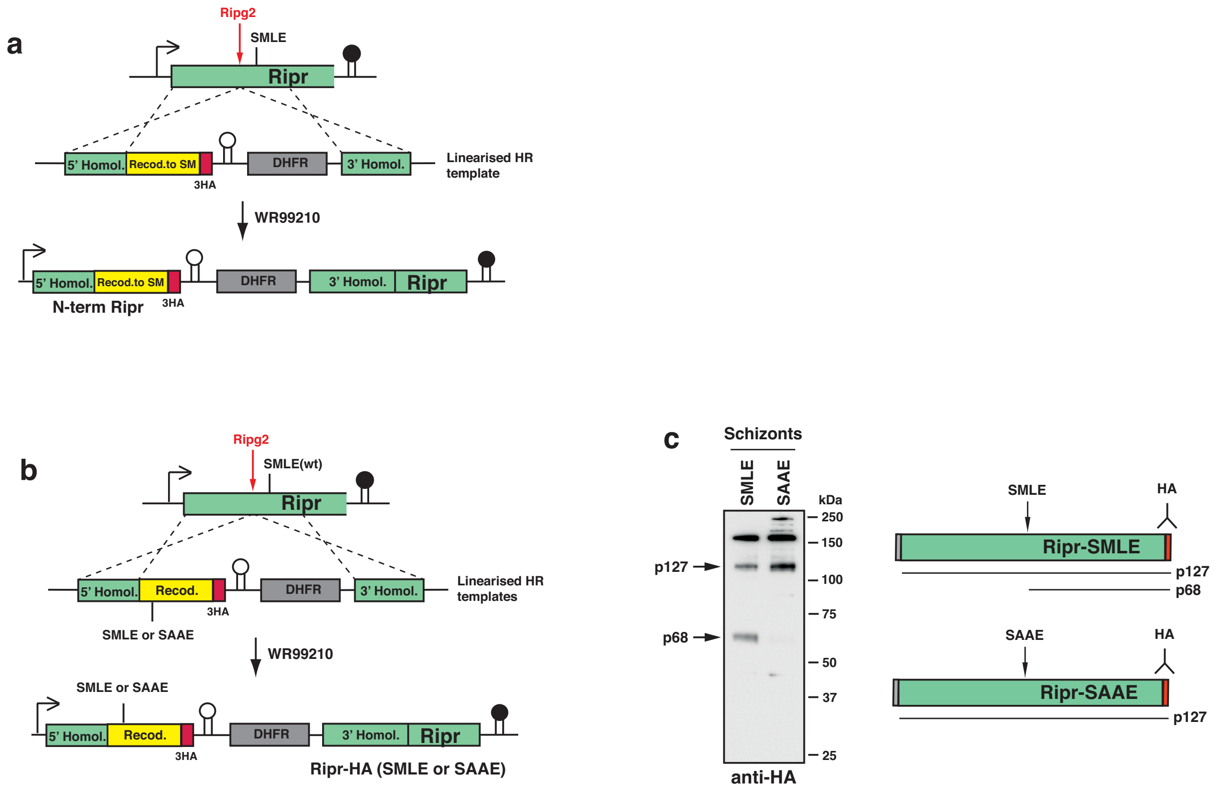

Figure S7. Investigating PMX processing of PfRipr. a. The PfRipr N-terminal protein up to the PMX site was insufficient to allow parasite viability. A homology-directed repair (HR) construct that would produce a parasite encoding a HA-tagged version of the N-terminal PfRipr sequence, up to the PMX site, was constructed. The PMX site in PfRipr is 'SMLE', so the recodoned gene was synthesized up to and including the 'SM' sequence (labelled 'Recod. to SM' in the schematic). No viable parasites were obtained despite multiple attempts. **b.** Generation of a C-terminally tagged PfRipr-HA parasite with a wild type PMX cleavage site (SMLE) and a mutated cleavage site (SAAE). The right-pointing arrow is the endogenous *pfripr* promoter, the black filled-in stalk is the endogenous *pfripr* terminator and the unfilled stalk depicts the *P.berghei dhfr-ts* terminator. **c**, Proteins from synchronised schizonts of the SMLE and SAAE parasites were blotted, then probed with an anti-HA mAb. A schematic representation of the expected products following PMX cleavage of both proteins is shown at right.

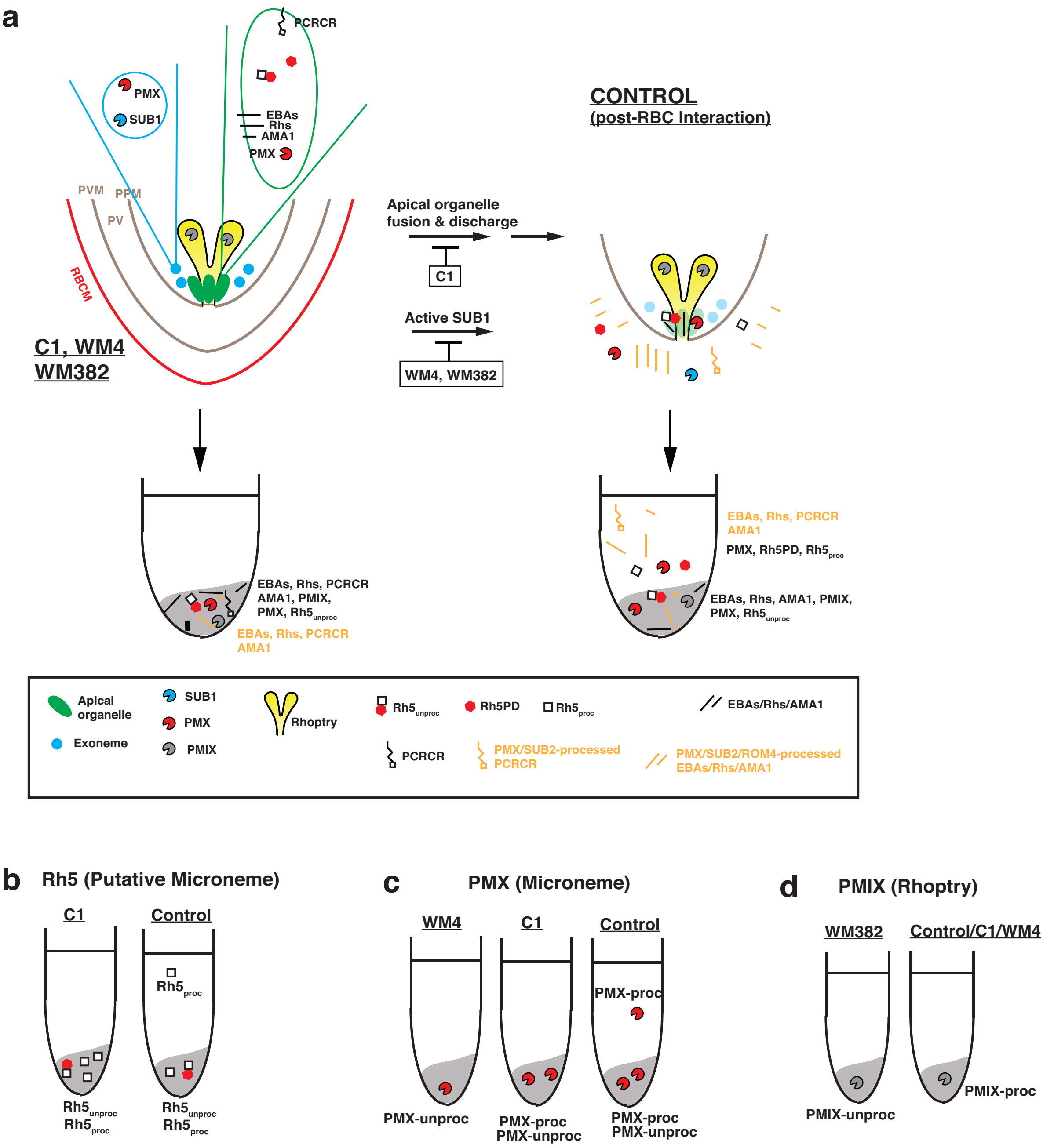

Figure S8. Fractionation of proteins into Supernatant and Merozoite compartments in Processing Inhibition Assays (PIA). a. A diagram of a merozoite with its parasite plasma membrane (PPM), trapped within the parasitophorous vacuole membrane (PVM) and red blood cell membrane (RBCM), following treatment with C1, WM4 or WM382. At right is a diagram of a merozoite that has egressed and interacted with a RBC (Control). The various proteins and organelles are summarised in the Box below. Below the C1/WM4/WM382 and Control conditions is a diagram of where various proteins are located following fractionation of Processing Inhibition Assay samples into supernatant and merozoite fractions, following a spin at 10000 g /10 min. **b-d.** The fractionation of merozoite proteins in various compartments such as PfRh5 (putative microneme), PMIX (rhoptry) and PMX (microneme) under different treatments are shown. The PIA treatments are Compound 1 (C1), WM4 and WM382. For each protein, the expected fractionation into either supernatant or merozoite compartment following

centrifugation at 10,000 g /10 min, is shown in the schematic. For each protein, 'unproc' indicates unprocessed and 'proc' indicates processed. For the PfRh5 protein, unprocessed and processed versions are called $Rh5_{unproc}$ and $Rh5_{proc}$ respectively.

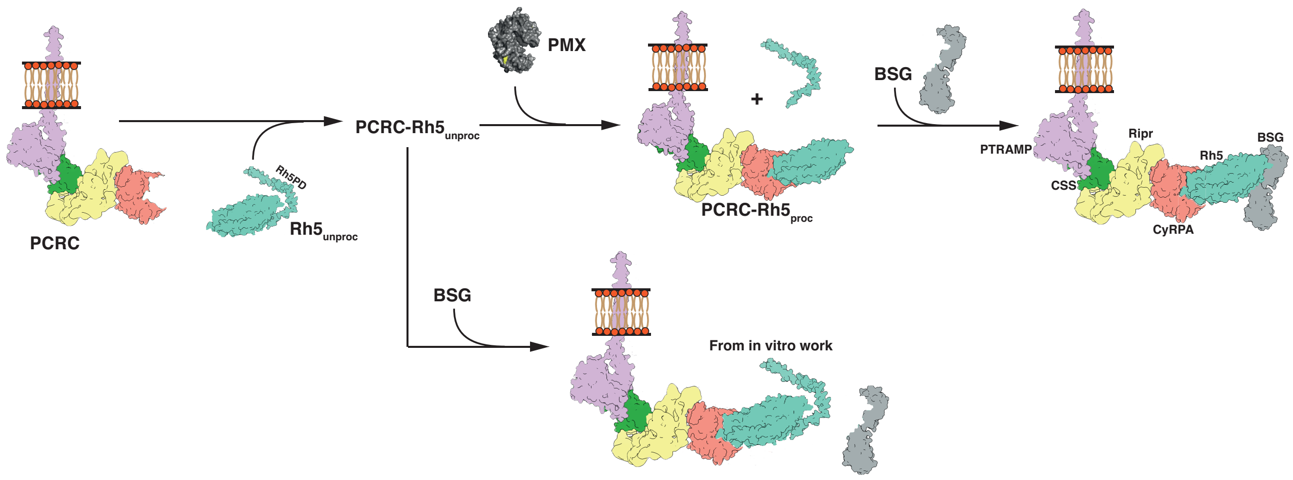

Figure S9. Model for the role of PfRh5 in PCRCR formation. The known structures of CSS (PDB ID: 7UNZ), PfRipr, CyRPA, PfRh5 (PDB ID: 6MPV), basigin (BSG) (PDB ID: 3B5H), Plasmepsin X (PMX) (PDB ID: 7TBD) and the AlphaFold Monomer v2.0 structures of PTRAMP (Uniprot: Q8I5M8) and Rh5PD (UniProt: Q8IFM5) are shown. The approximate location of PTRAMP inserted into the merozoite membrane is shown. Unprocessed PfRh5 shown as $Rh5_{unproc}$. PMX processing of PCRC-$Rh5_{unproc}$ produces PCRC-$Rh5_{proc}$.

Supplementary Tables

Table S1. *P. falciparum* parasites and description of transgenic changes.

Parasite name	Description/Details	Source
3D7	Parental line for direct CRISPR-cas9 manipulations	David Walliker (Uni of Edinburgh)
Pfs47DiCre	3D7 line where DiCre is integrated into Pfs47 locus	[2]
Rh5-iKO	Rh5-HA in Pfs47DiCre (ie. Rh5 inducible KO). Parental line for subsequent strain generation	[3]
Rh5-NFLQ	wt Rh5 (NFLQ) with C-term HA, in 3D7	This study
Rh5-NFLA	Mutant Rh5 (NFLA) with C-term HA, in 3D7	This study
Rh5-AFLA	Mutant Rh5 (AFLA) with C-term HA, in 3D7	This study
Rh5-NFAA	Mutant Rh5 (NFAA) with C-term HA, in 3D7	This study
Rh5-NALA	Mutant Rh5 (NALA) with C-term HA, in 3D7	This study
Rh5PD-CHA	Rh5PD tagged with C-term HA, in 3D7	This study
Rh5PD-NHA	Rh5PD tagged with N-term HA, in 3D7	This study
Rh5-HA	wt Rh5 with C-term HA, in 3D7. Parental line for subsequent strain generation	This study
RiprHA-SMLE	wt Ripr (SMLE) with C-term HA, in 3D7	This study
RiprHA-SAAE	Mutant Ripr (SAAE) with C-term HA, in 3D7	This study
Sel1-iKO	Sel1 with C-term HA, truncated with DiCre/Rapa, in Pfs47DiCre	This study
P113-iKO	P113 with C-term HA, truncated with DiCre/Rapa, in Pfs47DiCre	This study
GRP170-iKO	GRP170 with C-term HA, truncated with DiCre/Rapa, in Pfs47DiCre	This study
10TM-iKO	10TM with C-term HA, truncated with DiCre/Rapa, in Pfs47DiCre	This study
Rh5HA/Rh5DPDnG	Rh5HA re-CRISPR with Rh5DPD nGreen	This study
Rh5HA/Rh5nG	Rh5HA re-CRISPR with Rh5 nGreen	This study
Rh5iKO/Rh5DPDcomp	Rh5-iKO re-CRISPR with Rh5DPD-FLAG	This study
Rh5iKO/Rh5-NAAQ	Rh5-iKO re-CRISPR with Rh5-NAAQ-FLAG	This study
Rh5iKO/Rh5-NFLQ	Rh5-iKO re-CRISPR with Rh5-NFLQ-FLAG	This study
Rh5iKO/Rh5-tmut	Rh5-iKO re-CRISPR with Rh5tmut-FLAG	This study

Table S2. Sequence of oligonucleotides for plasmid constructs.

Plasmid	Fig.	Sequence (5' to 3')	Name	Function
Rh5-NFLQ<br>Rh5-NFLA<br>Rh5-AFLA<br>Rh5-NFAA<br>Rh5-NALA<br>Rh5PD-CHA	7	TAAGTATATAATATTtaatgaagactataaaaatgGTTTTAGAGCTAGAA<br>TTCTAGCTCTAAAACcatttttatagtcttcattaAATATTATATACTTA<br>Agctgcggccgccatgaagaattgagtcacataatag<br>agcttacgtagaatgatctttagcattatttgtttttatattc<br>agctgaattctaaaaatgttaattttttacaatatc<br>agctggtaccttttattaaaatcattctcatgg	TT1242<br>TT1243<br>TT1238<br>TT1239<br>TT1240<br>TT1241	Rh5g2 guide<br>Rh5g2 guide<br>PfRh5 5' flank<br>PfRh5 5' flank<br>PfRh5 3' flank<br>PfRh5 3' flank
Rh5ΔPD<br>Rh5PD-NHA	Supp.<br>Fig. 4<br>Supp.<br>Fig. 6	TAAGTATATAATATTtttgaatttactatatgtacGTTTTAGAGCTAGAA<br>TTCTAGCTCTAAAACgtacatatagtaaattcaaaAATATTATATACTTA<br>agctgcggccgcgtttttacaccaattaaacaatggt<br>agcttacgtatataagtatttatttatgtatctaca<br>agctgaattcaagttttgaaaatgcaataaaaaaaacga<br>agctacgcgtcactttccatatgtagaggacttatga	TT1280<br>TT1281<br>TT1276<br>TT1277<br>TT1278<br>TT1279	Rh5g3 guide<br>Rh5g3 guide<br>PfRh5 5' flank<br>PfRh5 5' flank<br>PfRh5 3' flank<br>PfRh5 3' flank
Rh5-HA<br>Rh5-iKO	Supp.<br>Fig.1<br>Supp.<br>Fig.2<br>Supp.<br>Fig.5	TAAGTATATAATATTgacagatgatgaaaccgaagGTTTTAGAGCTAGAA<br>TTCTAGCTCTAAAACcttcggtttcatcatctgtcAATATTATATACTTA<br>Agctgcggccgccgtgctatataaacatatttacg<br>Tcagatttatcatcgatttc<br>Agctgaattcgaagatagtatacaagatac<br>agctccatggatattcatatacataccatg	TT685<br>TT686<br>TT683<br>TT684<br>TT681<br>TT682	Rh5g1 guide<br>Rh5g1 guide<br>PfRh5 5' flank<br>PfRh5 5' flank<br>PfRh5 3' flank<br>PfRh5 3' flank
N-term Ripr<br>RiprHA-SMLE<br>RiprHA-SAAE	Supp.<br>Fig.7	TAAGTATATAATATTgggaaaaccaaatgtgcttaGTTTTAGAGCTAGAA<br>TTCTAGCTCTAAAACtaagcacatttggttttcccAATATTATATACTTA<br>agctgcggccgcaatgatgatacacataaagcaa<br>agctcttaagtgattctgtaacatatttcaatttg<br>agctggtacctacaatagatccaatagatgattt<br>agctctgcagtatcaaaatggtatattaaacca	TT1318<br>TT1319<br>TT1314<br>TT1315<br>TT1316<br>TT1317	Ripg2 guide<br>Ripg2 guide<br>Ripr 5' flank<br>Ripr 5' flank<br>Ripr 3' flank<br>Ripr 3' flank
Sel1-iKO	3	TAAGTATATAATATTtaaggaattgaagaataaggGTTTTAGAGCTAGAA<br>TTCTAGCTCTAAAACccttattcttcaattccttaAATATTATATACTTA<br>agctgcggccgcAAAGCAGCTGATAAAGGTGATAAT<br>agctcttaagATTCTCCACAATATTGTATTTTTC<br>agctgaattcAAACAATATTCACAATGTATATAATAA<br>agctGGTACCTTACATTCTCTTTTCTATTCACATG	TT1338<br>TT1339<br>TT1334<br>TT1335<br>TT1336<br>TT1337	Selg1 guide<br>Selg1 guide<br>Sel1 5' flank<br>Sel1 5' flank<br>Sel1 3' flank<br>Sel1 3' flank
P113-iKO	3	TAAGTATATAATATTgaacaagatgaaaatgttaaGTTTTAGAGCTAGAA<br>TTCTAGCTCTAAAACttaacattttcatcttgttcAATATTATATACTTA<br>agctgcggccgcacattgtgaagttcaatgtttga<br>agctccatggaactagaatctgtacttt<br>agctggtaccgagttcatcacaagaaatgaattc<br>agctctgcagtctatagtatctattttattcattg	TT1326<br>TT1327<br>TT1322<br>TT1323<br>TT1324<br>TT1325	P113g1 guide<br>P113g1 guide<br>P113 5' flank<br>P113 5' flank<br>P113 3' flank<br>P113 3' flank
GRP170-iKO	3	TAAGTATATAATATTgaagaacaaaaagatggagcGTTTTAGAGCTAGAA<br>TTCTAGCTCTAAAACgctccatctttttgttcttcAATATTATATACTTA<br>agctgcggccgcAAAGGAGGATTTCAATTAAAGGA<br>agctcttaagATACCAAATTTATCTAAATGAAAT<br>agctgaattcACAAAAGATAACAAAAAAGAAGGTGA<br>agctctgcagTTCAGCTCTTTCTTTGATTGGA	TT1332<br>TT1333<br>TT1328<br>TT1329<br>TT1330<br>TT1331	GRPg1 guide<br>GRPg1 guide<br>GRP 5' flank<br>GRP 5' flank<br>GRP 3' flank<br>GRP 3' flank
10TM-iKO	3	TAAGTATATAATATTtatgattgatgagcacaatgGTTTTAGAGCTAGAA<br>TTCTAGCTCTAAAACcattgtgctcatcaatcataAATATTATATACTTA<br>agctgcggccgcATGATTGAAGAATCACATAATTTGA<br>agctAGATCTTTTTTTGATAATCACATTGTTGT<br>agctgaattcCCCAATTTGAACATAAAATTCTTAAT<br>agctctgcagAAAATATCCTTATAATTTTCATGACT	TT1344<br>TT1345<br>TT1340<br>TT1341<br>TT1342<br>TT1343	10TMg1 guide<br>10TMg1 guide<br>10TM 5' flank<br>10TM 5' flank<br>10TM 3' flank<br>10TM 3' flank
Rh5HA /<br>Rh5ΔPDnG<br>Rh5HA /<br>Rh5nG<br>Rh5iKO /<br>Rh5ΔPDcomp<br>Rh5iKO / Rh5-<br>NAAQ<br>Rh5iKO<br>Rh5-NFLQ<br>Rh5iKO<br>Rh5-tmut	Supp.<br>Fig. 1<br>Supp.<br>Fig. 2	TAAGTATATAATATTgaaaaacctaaagctgttatGTTTTAGAGCTAGAA<br>TTCTAGCTCTAAAACataacagctttaggtttttcAATATTATATACTTA<br>agctacgcgtggaaaataggacacaacaaaatgt<br>agctgaattcttatagatataaatattttatttcccatatcatcatattct<br>agctcttaagaatatatcatctagtcgttcttctg<br>agctgcggccgccaagctaaacctattaacatatcat	TT1196<br>TT1197<br>TT1298<br>TT1299<br>TT1300<br>TT1301	P230g2 guide<br>P230g2 guide<br>P230p 5' flank<br>P230p 5' flank<br>P230p 3' flank<br>P230p 3' flank
Rh5HA<br>Rh5ΔPDnG<br>Rh5HA Rh5nG<br>Rh5iKO<br>Rh5ΔPDcomp<br>Rh5iKO<br>Rh5-<br>NAAQ<br>Rh5iKO<br>Rh5-NFLQ<br>Rh5iKO<br>Rh5-tmut	Supp.<br>Fig. 1<br>Supp.<br>Fig. 2	Agcttacgtaactcaattcttcatgggtgtataatttatt<br>agctcttaagggtacattttgctctatattttgtt	TT1303<br>TT1305	Rh5 promoter<br>Rh5 promoter

Table S3. Plasmids used for assembling constructs.

Name	Use	Source
pUF-cas9G	Plasmid for cloning annealed guide oligos	[4]
p1.2	Parent plasmid used to tag genes with 3' HA/StrepII	[5]
NABS4	p1.2 derivative, to tag genes with 3' HA. For HDR constructs. *hdhfr* marker.	This study
BNABS4	NABS4 derivative, to tag genes with 3' FLAG. *bsd* marker.	This study
Rh5-HA	Rh5-HA in p1.2	This study
Rh5-NFLQ, Rh5-NFLA, Rh5-AFLA, Rh5-NFAA, Rh5-NALA	Rh5HA mutant plasmids in NABS4	This study
Rh5PD-CHA, Rh5PD-NHA, Rh5DPD	HA-tagged prodomain or deleted PD in NABS4	This study
Nterm Ripr, RiprHA-SMLE, RiprHA-SAAE	HA-tagged Ripr variants in NABS4	This study
Sel1-iKO, P113-iKO, GRP170-iKO, 10TM-iKO	Inducible KO plasmids in NABS4	This study
Rh5DPDnG, Rh5nG	Re-CRISPR plasmids in BNABS4 (*bsd*)	This study
Rh5PDcomp, Rh5-NAAQ, Rh5-NFLQ, Rh5-tmut	Re-CRISPR plasmids in BNABS4 (*bsd*)	This study

Table S4. Antibodies used in this study.

Name	Species	Cat. No.	Source	IFA dilution	Western blot dilution
anti-HA (3F10)	Rat	11867423001	Roche	1/300	-
HRP anti-HA (3F10)	Rat	12013819001	Roche	-	1/1000
anti-rabbit IgG - HRP	Goat	AP187P	Merck	-	1/4000
anti-mouse IgG - HRP	Goat	AP124P	Merck	-	1/1000
anti-mouse IgG - AlexaFluor488	Goat	A11001	Invitrogen	1/1000	-
anti-rat IgG - AlexaFluor594	Goat	A11007	Invitrogen	1/1000	-
anti-nGreen	Mouse	32f6	Chromotek	1/300	-
anti-FLAG - HRP	Mouse	A8592	Sigma	-	1/1000
anti-hsp70	Rabbit	-	[6]	-	1/4000
anti-PTRAMP (1D9)	Mouse	-	[3]	-	5 µg/ml
anti-CSS (2D2)	Rat	-	[3]	-	5 µg/ml
anti-CyRPA (7A6)	Mouse	-	[7]	-	
anti-Ripr (1G12)	Mouse	-	[3]	-	5 µg/ml
anti-Rh5 (5A9)	Mouse	-	[3]	-	5 µg/ml
anti-Rh5 (6H2)	Mouse	-	[5]	-	5 µg/ml
anti-EBA140 (1H5)	Mouse	-	[8]	-	10 µg/ml
anti-AMA1	Rabbit	-	[9]	-	1/1000
anti-Rh2 (6F12)	Mouse	-	[10]	-	20 µg/ml

Table S5. Chemicals used in this study.

Chemical/Reagent	Reference	Source
Amersham Protran 0.2 µm Nitrocellulose	10600001	GE Healthcare
Restriction enzymes	-	New England Biolabs
WM4, WM382	-	[8]
Compound 1 (C1)	-	In house WEHI synthesis

Table S6. Software used in this study.

Software	Version	Source
Prism	9.3.1	www.graphpad.com/
ImageLab	6.1.0 build 7	BioRad Laboratories Inc.
FlowJo for Mac	10.7	Becton Dickinson
SnapGene	1.6.17	Dotmatics
Labarchives	-	Dotmatics
MaxQuant	1.6.17	Max Planck Institute
ImageJ2	Version 2.9.0/1.54b	-

Supplementary References

1. Scally SW*, et al.* PCRCR complex is essential for invasion of human erythrocytes by *Plasmodium falciparum*. *Nat Microbiol* **7**, 2039-2053 (2022).

2. Knuepfer E, Napiorkowska M, van Ooij C, Holder AA. Generating conditional gene knockouts in *Plasmodium* - a toolkit to produce stable DiCre recombinase-expressing parasite lines using CRISPR/Cas9. *Scientific reports* **7**, 3881 (2017).

3. Scally SW*, et al.* PCRCR complex is essential for invasion of human erythrocytes by *Plasmodium falciparum. Nat Microbiol* **7**, 2039-2053 (2022).

4. Volz JC*, et al.* Essential Role of the PfRh5/PfRipr/CyRPA Complex during *Plasmodium falciparum* Invasion of Erythrocytes. *Cell Host Microbe* **20**, 60-71 (2016).

5. Baum J*, et al.* Reticulocyte-binding protein homologue 5 - an essential adhesin involved in invasion of human erythrocytes by *Plasmodium falciparum. Int J Parasitol* **39**, 371-373 (2009).

6. Bianco AE*, et al.* A repetitive antigen of *Plasmodium falciparum* that is homologous to heat shock protein 70 of Drosophila melanogaster. *Proc Natl Acad Sci USA* **83**, 8713-8717 (1986).

7. Chen L*, et al.* Structural basis for inhibition of erythrocyte invasion by antibodies to *Plasmodium falciparum* protein CyRPA. *eLife* **6**, (2017).

8. Favuzza P*, et al.* Dual Plasmepsin-Targeting Antimalarial Agents Disrupt Multiple Stages of the Malaria Parasite Life Cycle. *Cell Host Microbe* **27**, 642-658 e612 (2020).

9. Coley AM*, et al.* Rapid and precise epitope mapping of monoclonal antibodies against *Plasmodium falciparum* AMA1 by combined phage display of fragments and random peptides. *Protein Eng* **14**, 691-698 (2001).

10. Triglia T*, et al. Plasmodium falciparum* merozoite invasion is inhibited by antibodies that target the PfRh2a and b binding domains. *PLoS Pathog* **7**, e1002075 (2011).
