# Supplementary material for: Plasmepsin X activates the PCRCR complex of Plasmodium falciparum by processing PfRh5 for erythrocyte invasion
Source: Nat Commun. 2023 Apr 19;14:2219. doi: 10.1038/s41467-023-37890-2 (PMC10113190; doi:10.1038/s41467-023-37890-2)
Supplement: Supplementary file 3 — Reporting Summary [file 41467_2023_37890_MOESM3_ESM.pdf]

nature portfolio

Corresponding author(s): Alan F. Cowman

Last updated by author(s): Mar 24, 2023

Reporting Summary

Nature Portfolio wishes to improve the reproducibility of the work that we publish. This form provides structure for consistency and transparency in reporting. For further information on Nature Portfolio policies, see our Editorial Policies and the Editorial Policy Checklist.

Statistics

For all statistical analyses, confirm that the following items are present in the figure legend, table legend, main text, or Methods section.

n/a	Confirmed	
☐	☒	The exact sample size (*n*) for each experimental group/condition, given as a discrete number and unit of measurement
☐	☒	A statement on whether measurements were taken from distinct samples or whether the same sample was measured repeatedly
☐	☒	The statistical test(s) used AND whether they are one- or two-sided<br>*Only common tests should be described solely by name; describe more complex techniques in the Methods section.*
☒	☐	A description of all covariates tested
☒	☐	A description of any assumptions or corrections, such as tests of normality and adjustment for multiple comparisons
☐	☒	A full description of the statistical parameters including central tendency (e.g. means) or other basic estimates (e.g. regression coefficient) AND variation (e.g. standard deviation) or associated estimates of uncertainty (e.g. confidence intervals)
☐	☒	For null hypothesis testing, the test statistic (e.g. *F*, *t*, *r*) with confidence intervals, effect sizes, degrees of freedom and *P* value noted<br>*Give P values as exact values whenever suitable.*
☒	☐	For Bayesian analysis, information on the choice of priors and Markov chain Monte Carlo settings
☒	☐	For hierarchical and complex designs, identification of the appropriate level for tests and full reporting of outcomes
☒	☐	Estimates of effect sizes (e.g. Cohen's *d*, Pearson's *r*), indicating how they were calculated

Our web collection on statistics for biologists contains articles on many of the points above.

Software and code

Policy information about availability of computer code

Data collection	MaxQUANT (version 1.6.17).
Data analysis	Image Lab 6.1, Biorad. ImageJ version 2.9.0/1.54b. MaxQuant (version 1.6.17). GraphPad Prism v 9.3.1.

For manuscripts utilizing custom algorithms or software that are central to the research but not yet described in published literature, software must be made available to editors and reviewers. We strongly encourage code deposition in a community repository (e.g. GitHub). See the Nature Portfolio guidelines for submitting code & software for further information.

Data

Policy information about availability of data

All manuscripts must include a data availability statement. This statement should provide the following information, where applicable:

- Accession codes, unique identifiers, or web links for publicly available datasets
- A description of any restrictions on data availability
- For clinical datasets or third party data, please ensure that the statement adheres to our policy

The mass spectrometry proteomics data have been deposited to the ProteomeXchange Consortium via the PRIDE partner repository with the dataset identifier PXD039646 (Username: reviewer_pxd039646@ebi.ac.uk, Password: pdJIA5kW). P. falciparum sequences were derived from PlasmoDB (https://plasmodb.org/plasmo/app). The datasets generated during and/or analysed during the current study are available from the corresponding author on reasonable request. Structures used are CSS (PDB ID: 7UNZ), PfRipr, CyRPA, PfRh5 (PDB ID: 6MPV), basigin (BSG) (PDB ID: 3B5H).

Human research participants

Policy information about studies involving human research participants and Sex and Gender in Research.

Reporting on sex and gender	Not applicable
Population characteristics	Not applicable
Recruitment	Not applicable
Ethics oversight	Not applicable

Note that full information on the approval of the study protocol must also be provided in the manuscript.

Field-specific reporting

Please select the one below that is the best fit for your research. If you are not sure, read the appropriate sections before making your selection.

☒ Life sciences ☐ Behavioural & social sciences ☐ Ecological, evolutionary & environmental sciences

For a reference copy of the document with all sections, see nature.com/documents/nr-reporting-summary-flat.pdf

Life sciences study design

All studies must disclose on these points even when the disclosure is negative.

Sample size	No statistical method was used to predetermine sample size. Instead, sample sizes were chosen according to best practices in the field and previous studies (Wong et al. Nature 2019, Scally et al. Nat Micro 2022).
Data exclusions	No data were excluded.
Replication	Monitoring of P. falciparum parasitemia of the inducible knockdowns was performed in duplicate in two independent experiments. All attempts at replication were successful.
Randomization	Randomization was not relevant to this study as no subjective judgements were required about which data to include, exclude or measure.
Blinding	The investigators were not blinded to the group allocation during the experiment and/or when assessing the outcome.

Reporting for specific materials, systems and methods

We require information from authors about some types of materials, experimental systems and methods used in many studies. Here, indicate whether each material, system or method listed is relevant to your study. If you are not sure if a list item applies to your research, read the appropriate section before selecting a response.

Materials & experimental systems

n/a	Involved in the study	
☐	☒	Antibodies
☐	☒	Eukaryotic cell lines
☒	☐	Palaeontology and archaeology
☒	☐	Animals and other organisms
☒	☐	Clinical data
☒	☐	Dual use research of concern

Methods

n/a	Involved in the study	
☒	☐	ChIP-seq
☒	☐	Flow cytometry
☒	☐	MRI-based neuroimaging

Antibodies

Antibodies used	Antibodies and monoclonal antibodies were raised in rabbits, mice or rats and all procedures approved by the Walter and Eliza Hall Institute of Medical Research Animal Ethics Committee.<br>In this study, we used the following antibodies: rat mAb, anti-HA (Roche 3F10, Cat.: 11867423001); rat mAb, HRP anti-HA (Roche 3F10, Cat.: 12013819001); mouse mAb, 1D9 anti-PfPTRAMP (Scally et al, 2022); rat mAb 2D2 anti-PfCSS (Scally et al, 2022); mouse mAb 7A6 anti-CyRPA (Chen et al, Elife 2017); mouse mAb 1G12 anti-Ripr (Scally et al, 2022); mouse mAbs 5A9 and 6H2 anti-PfRh5 (Scally et al, 2022); mouse mAb, anti-nGreen (Chromotek, Cat.: 32f6); mouse mAb, HRP anti-FLAG (Sigma, Cat.: A8592); rabbit anti-

hsp70 pAb (Bianco et al, 1986; PNAS 83:8713); mouse mAb 1H5 anti-EBA140 (Favuzza et al, 2020); Rabbit anti-AMA1 pAb (Coley et al, 2001); mouse mAb, 6F12 anti-Rh2 (Triglia et al, 2011).
The following secondary antibodies labelled with Alexa 488/594 fluorophores (Life Technologies) and HRP antibodies (Merck) were used: goat anti-mouse 488 (Cat.: A11001), goat anti-rat 594 (Cat.: A11007), goat anti-rabbit-HRP (Cat.: AP187P), goat anti-mouse-HRP (AP124P).

Validation

Rat mAbs, anti-HA and HRP anti-HA validated by the supplier by western blot.
Goat anti-mouse 488 validated by the supplier by IFA.
Goat anti-rat 594 validated by the supplier by IFA.
Goat anti-rabbit-HRP validated by the supplier by western blot.
Goat anti-mouse-HRP validated by the supplier by western blot.
Mouse mAb anti-nGreen validated by the supplier by IFA and ELISA.
Mouse mAb anti-FLAG-HRP validated by the supplier by western blot.

Mouse mAb 1D9 anti-PTRAMP and Rat mAb 2D2 anti-CSS, validated by western blot in Scally et al, 2022.
Mouse mAb 7A6 anti-CyRPA validated by western blot in Chen et al, 2017.
Mouse mAb 1G12 anti-Ripr validated by western blot in Scally et al, 2022.
Mouse mAb 5A9 anti-Rh5 validated by western blot in Scally et al, 2022.
Mouse mAb 6H2 anti-Rh5 validated by western blot in Baum et al, 2009.
Mouse mAb 1H5 anti-EBA140 validated by western blot in Favuzza et al, 2020.
Rabbit pAb anti-AMA1 validated by western blot in Coley et al, 2001.
Mouse mAb 6F12 anti-Rh2 validated by western blot in Triglia et al, 2011.
Rabbit pAb anti-hsp70 validated by western blot in Bianco et al, 1986; PNAS 83:8713.

Eukaryotic cell lines

Policy information about cell lines and Sex and Gender in Research

Cell line source(s)

3D7 P. falciparum line
O+ erythrocyte (Australian red-cross bloodbank, South Melbourne, Australia)
Sf21 cells (ThermoFisher Scientific)

Authentication

The P. falciparum lines are periodically sequenced for other projects. This serves as an authentication that they are the expected versions of the P. falciparum lines. Sf21 cell lines were purchased or obtained with the certificate of analysis.

Mycoplasma contamination

All cells lines are tested periodically for Mycoplasma infection and were negative.

Commonly misidentified lines
(See ICLAC register)

N/A
